# Structure of a filamentous virus uncovers familial ties within the archaeal virosphere

**DOI:** 10.1093/ve/veaa023

**Published:** 2020-04-29

**Authors:** Fengbin Wang, Diana P Baquero, Zhangli Su, Tomasz Osinski, David Prangishvili, Edward H Egelman, Mart Krupovic

**Affiliations:** v1 Department of Biochemistry and Molecular Genetics, University of Virginia, PO Box 800733, Charlottesville, VA 22908, USA; v2 Archaeal Virology Unit, Department of Microbiology, Institut Pasteur, 25 Rue du Dr Roux, Paris 75015, France; v3 Sorbonne Universités, Collège Doctoral, 7 Quai Saint-Bernard, Paris 75005, France; v4 Ivane Javakhishvili Tbilisi State University, 1 Chavchavadze Avenue, Tbilisi 0179, Georgia

**Keywords:** cryo-EM, hyperthermophilic archaea, major capsid proteins, virus evolution, virus structure

## Abstract

Viruses infecting hyperthermophilic archaea represent one of the most enigmatic parts of the global virome, with viruses from different families showing no genomic relatedness to each other or to viruses of bacteria and eukaryotes. Tristromaviruses, which build enveloped filamentous virions and infect hyperthermophilic neutrophiles of the order Thermoproteales, represent one such enigmatic virus families. They do not share genes with viruses from other families and have been believed to represent an evolutionarily independent virus lineage. A cryo-electron microscopic reconstruction of the tristromavirus Pyrobaculum filamentous virus 2 at 3.4 Å resolution shows that the virion is constructed from two paralogous major capsid proteins (MCP) which transform the linear dsDNA genome of the virus into A-form by tightly wrapping around it. Unexpectedly, the two MCP are homologous to the capsid proteins of other filamentous archaeal viruses, uncovering a deep evolutionary relationship within the archaeal virosphere.

## 1. Introduction

Systematic comparison of sequences and structures of the major (nucleo)capsid proteins (MCP) from across the virosphere has shown that viruses from ∼80 per cent of known virus families can be categorized into seventeen architectural classes represented by unique MCP folds (Krupovic and Koonin 2017). However, the remaining ∼20 per cent of virus groups encode MCPs for which the structural fold is not known and could not be predicted using state-of-the-art sequence analyses, representing the ‘unknown’ of the virosphere and potentially concealing novel architectural solutions for virion organization. Notably, ∼33 per cent of this structural ‘dark matter’ corresponds to viruses infecting archaea. Filamentous archaeal viruses, all infecting hyperthermophilic hosts growing optimally at 80°–90 °C, have been classified into four families: *Clavaviridae*, *Rudiviridae*, *Lipothrixviridae* and *Tristromaviridae* (Prangishvili et al. 2017). High-resolution structures are available for representatives of the first three virus families and display MCP folds unprecedented among bacterial and eukaryotic viruses (Krupovic and Koonin 2017; Hartman et al. 2019). Clavavirus APBV1 infects *Aeropyrum pernix* (marine hyperthermophile; order Desulfurococcales) and encodes one of the simplest known MCPs, folded as two α-helices connected by a β-hairpin. Approximately 1,000 copies of the MCP form a hollow cylindrical virion into which the circular dsDNA genome is packed (Ptchelkine et al. 2017). In contrast, virions of rudiviruses (DiMaio et al. 2015) and lipothrixviruses (Kasson et al. 2017; Liu et al. 2018), which infect members of the order Sulfolobales (acidophilic hyperthermophiles), are formed by condensation of a linear dsDNA genome by homodimeric (in rudiviruses) or heterodimeric paralogous (in lipothrixviruses) MCPs displaying a C-terminal four-helix bundle fold with an extended N-terminal α-helical arm. The α-helical arm wraps tightly around the DNA, transforming the dsDNA into the A-form. Whereas virions of rudiviruses are fairly rigid and non-enveloped, those of lipothrixviruses are more flexible and covered with a lipid envelope containing other viral proteins. Despite these differences, rudiviruses and lipothrixviruses share extensive gene content and are thus unified into an order *Ligamenvirales* (Prangishvili and Krupovic 2012). Tristromaviruses infect neutrophilic hyperthermophiles of the order Thermoproteales. Although the high-resolution structure of tristromaviruses is not known, biochemical studies have shown that virions are constructed from three major structural proteins, with MCP1 and MCP2 forming a helical nucleocapsid, and VP3 being associated with an external lipid membrane (Rensen et al. 2016). Due to lack of detectable protein and nucleotide sequence similarity between tristromaviruses and those of other known viruses, tristromaviruses were suggested to represent an evolutionarily independent virus lineage (Iranzo et al. 2016). To study the virion organization of tristromaviruses, we have determined the structure of the Pyrobaculum filamentous virus 2 (PFV2) at 3.4 Å resolution.

## 2. Materials and methods

### 2.1 PFV2 production and purification

For virus production, 150-ml culture of exponentially growing *Pyrobaculum arsenaticum* 2GA cells (Rensen et al. 2016) was infected with a fresh preparation of PFV2 (Baquero et al. 2020) at a multiplicity of infection of ∼0.1–1. The infected culture was incubated at 90 °C for 4 days without shaking. Following the removal of cells (7,000 rpm, 20 min, Sorvall 1500 rotor), the virions were collected and concentrated by ultracentrifugation (40,000 rpm, 2 h, 10 °C, Beckman 126 SW41 rotor). The concentrated virus particles were resuspended in buffer A (Quemin et al. 2015): 20 mM KH_2_PO_4_, 250 mM NaCl, 2.14 mM MgCl2, 0.43 mM Ca(NO_3_)_2_ and <0.001% trace elements of Sulfolobales medium, pH 6.

### 2.2 Cryo-electron micrograph image analysis and model building

The PFV2 sample (4.5 μl) was applied to discharged lacey carbon grids and plunge frozen using a Vitrobot Mark IV (FEI). Frozen grids were imaged in a Titan Krios at 300 keV and recorded with a Falcon III camera at 1.4 Å per pixel. Micrographs were collected using a defocus range of 1.25–2.25 μm, with a total exposure dose of 55 electrons/Å^2^ distributed into twenty-four fractions. To get an initial helical reconstruction volume, all of the micrographs were motion corrected using MotionCorr version 2.1 (Li et al. 2013), then used for contrast transfer function (CTF) estimation by the CTFFIND3 program (Mindell and Grigorieff 2003). After the images were corrected for the CTF through multiplication by the theoretical CTF, filament images amounting to ∼20 electrons/Å^2^ were extracted from dose-weighted fractions using the e2helixboxer program within EMAN2 (Tang et al. 2007). A small subset containing 30,000 overlapping 384-pixel-long segments (with a shift of ten pixels between adjacent subunits, ∼4 times the axial rise per subunit) was used to determine the helical symmetry in SPIDER (Shaikh et al. 2008), using IHRSR (Egelman 2000) after searching through a number of possible symmetries by trial and error. A ∼4.5 Å reconstruction was generated from this small subset, and this volume was subsequently filtered to 8 Å as the starting reference used in RELION (Zivanov et al. 2018). The micrographs and box coordinates of the full dataset were imported into RELION. After Refine3D, CTF refinement and Bayesian polishing, the final volume was estimated to have a resolution of 3.4 Å based on the map: map FSC, model: map FSC and d99 (Afonine et al. 2018) and was sharpened with a negative B factor of −155 Å^2^.

First, the density corresponding to a single MCP1 or MCP2 was segmented from the experimental filament density using Chimera (Pettersen et al. 2004). The full-length MCP1/MCP2 protein was built de novo into the segmented map using Rosetta CM (Wang et al. 2015), then adjusted manually in Coot (Emsley and Cowtan 2004) and real space refined in PHENIX (Afonine et al. 2018). Then EM density corresponding to A-DNA was segmented in Chimera, and A-DNA was manually put in the map and refined in PHENIX. Finally, the refined MCP1 and MCP2 single model were used to generate a filamentous model using the determined helical symmetry, and this filament model plus A-DNA were refined against the full cryo-electron micrograph (cryo-EM) map using PHENIX. MolProbity (Williams et al. 2018) was used to evaluate the quality of the filament model. The refinement statistics are shown in Table 1.

**Table 1.** Cryo-EM and refinement statistics for the PFV2.

Parameter	PFV2
Data collection and processing	
Magnification	59,000×
Voltage (kV)	300
Electron exposure (e^−^ Å^−2^)	20
Defocus range (μm)	−1.25 to −2.25
Pixel size (Å)	1.4
Symmetry imposed	Helical (rise: 2.86 Å; rotation: 22.95°)
Final particle images (*n*)	186,576
Map resolution (Å)	
‘Gold-standard’ map: map FSC (0.143)	3.4
Model: map FSC (0.38, which is √0.143)	3.4
d_99_	3.5
Refinement	
Initial model used (PDB code)	NA
Map-sharpening *B* factor (Å^2^)	−155
Root-mean-square deviations	
Bond lengths (Å)	0.008
Bond angles (°)	0.828
Validation	
MolProbity score	1.37
Clash score	4.0
Poor rotamers (%)	0
Ramachandran plot	
Favored (%)	96.9
Allowed (%)	3.1
Disallowed (%)	0

The PFV2 model used has the following IDs: Electron Microscopy Data Bank: 21094; PDB: 6V7B.

## 3. Results and discussion

**Figure 1.**
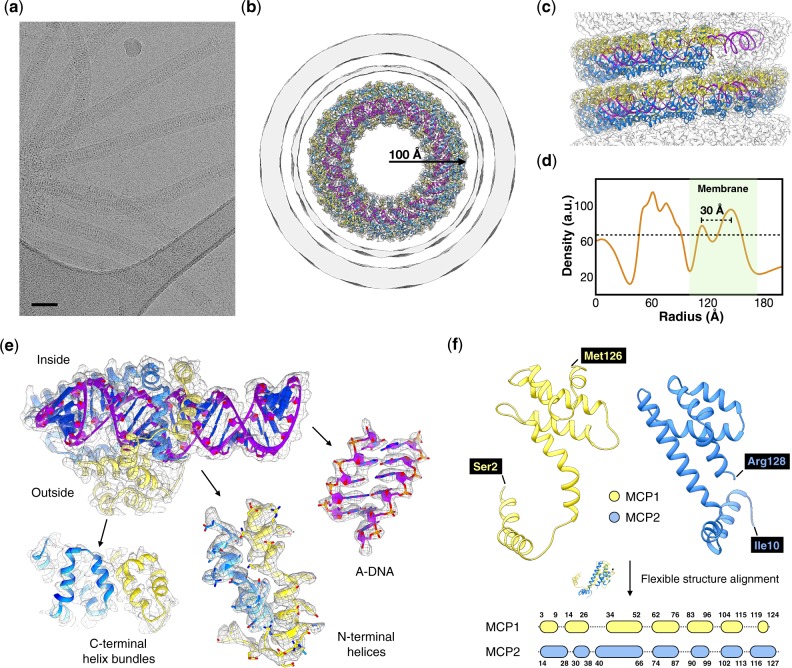
Cryo-EM of the PFV2. (a) Representative cryo-EM micrograph of the PFV2, taken from 5,458 micrographs recorded. Scale bar, 500 Å. (b, c) Three-dimensional reconstruction of PFV2 with atomic model built into the density. A top view (b) shows the nucleoprotein core and enveloping membrane. The membrane has been filtered to 8 Å due to the noise present from imposing the helical symmetry on an unstructured fluid. In the side view (c) the membrane has been removed. MCP1 and MCP2 subunits are in yellow and blue, respectively. A-form DNA is in purple. (d) The average radial density profile of the 3D reconstruction of PFV2. The area corresponding to the membrane is in green. The approximate threshold used for the surfaces shown in (b) is indicated by a dotted line. (e) Close-up views of the density map and fitted models of the MCPs and DNA. MCP1 is in yellow and MCP2 is in blue, while the DNA backbone is in magenta. (f) 3D alignment of MCP1 and MCP2. A flexible secondary structure alignment (bottom) reveals a one-to-one mapping of the local secondary structure elements between the two proteins.

Cryo-electron micrographs (Fig. 1a) show the membrane-enveloped virions of PFV2 to be ∼340 Å in diameter and ∼5,000 Å in length. The helical symmetry was determined to be a rise of 2.86 Å and a rotation of 22.95° per asymmetric unit, where the asymmetric unit contained a protein heterodimer and 12 bp (base pairs) of A-form DNA. The DNA, almost completely covered by the heterodimers (Fig. 1b and c), tightly supercoils in a solenoidal fashion with one right-handed supercoil per 45.4 Å turn of the capsid helix. The native twist of the DNA is therefore 11.3 bp/turn (564 bp in forty-seven local turns plus three supercoil turns). Notably, *Tristromaviridae* are the first family of neutrophilic and hyperthermophilic archaeal viruses in which genomic DNA adopts the A-form (all other known archaeal viruses in which the DNA was found to be A-form are acidophiles (DiMaio et al. 2015; Kasson et al. 2017; Liu et al. 2018; Wang et al. 2019)). This observation suggests that A-form DNA is a general adaptation of these viruses to extreme temperatures rather than pH.

The membrane (Fig. 1b and d) is seen as a thicker outer component and a thinner inner one. We attribute the thickened outer portion to the presence of the additional viral protein VP3. In the closely related PFV1 (97–99% pairwise sequence identity between the three major structural proteins, and 98.9% nucleotide identity over 70% of their genomes) (Baquero et al. 2020), VP3 was shown to remain associated with the membrane fraction when the envelope was removed (Rensen et al. 2016). However, we failed to see any order associated with this outer layer. In the membrane-enveloped AFV1 (Kasson et al. 2017) and SFV1 (Liu et al. 2018), the distance between the inner and outer membrane density peaks is ∼12 Å, the basis for models of a thin monolayer built from horseshoe-shaped lipids (Kasson et al. 2017). In contrast, this spacing is ∼30 Å in PFV2 suggesting that the lipids are arranged in a similar manner as in the host membrane, perhaps stabilized by VP3.

**Figure 2.**
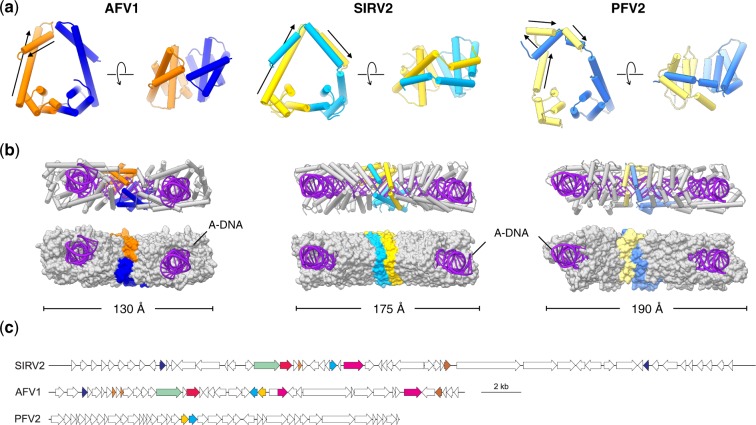
Comparison of the filamentous viruses AFV1, SIRV2 and PFV2. (a) MCP dimer (asymmetric unit) comparison of AFV1, SIRV2 and PFV2. The MCP1 of AFV1, SIRV2 and PFV2 are colored in orange, gold and yellow, respectively. The MCP2 of AFV1, SIRV2 and PFV2 are colored in blue, cyan and light blue, respectively. The N-terminal helices of MCP1 in AFV1, SIRV2 and PFV2 are marked with black arrows. (b) Wrapping of A-DNA in AFV1, SIRV2 and PFV2. Five MCP dimers are displayed: one MCP dimer is colored as in (a); the other four colored in gray. Proteins are shown in ribbon representation (top) and as surfaces (bottom). (c) Comparison of genome maps of rudivirus SIRV2 (NC_004086), lipothrixvirus AFV1 (NC_005830) and tristromavirus PFV2 (MN876844). Homologous genes (*E* < 1e–04) are indicated with the same colors. The homology between the MCPs of PFV2 (yellow and cyan ORFs) and those of the other two viruses is not recognizable by sequence similarity searches.

There was no ambiguity in fitting the sequences of MCP1 and MCP2 into the density map given the resolution achieved (Fig. 1e). While a 3D superposition of MCP1 and MCP2 shows significant differences in the overall folds, a flexible secondary structure alignment (Ye and Godzik 2003) reveals a one-to-one mapping of local secondary structure elements (Fig. 1f) indicative of obvious homology. Perhaps more surprisingly, the PFV2 heterodimer is quite similar to the homodimer of rudivirus SIRV2 (DiMaio et al. 2015) and the heterodimer of lipothrixvirus AFV1 (Kasson et al. 2017) (Fig. 2a and b), especially in the C-terminal four-helix bundle fold, whereas the three viruses share no apparent similarity either at the nucleotide or protein sequence level (Fig. 2c). Notably, PFV2 achieves a similar coverage of A-DNA as SIRV2 and AFV1 by swapping the upper half of the MCP1 N-terminal helix-arm to the other side of the MCP2 helix-arm (Fig. 2a and b).

Our results uncover an evolutionary relationship among three families of hyperthermophilic archaeal viruses, *Tristromaviridae*, *Rudiviridae* and *Lipothrixviridae*, including the homology of the MCPs and the similar compaction of the genomes as A-form DNA by these MCPs. The fact that, besides the structurally related MCPs, tristromaviruses and ligamenviruses (i.e. rudiviruses and lipothrixviruses) do not encode recognizable orthologs emphasizes the remarkable plasticity of archaeal virus gene content and suggest that the two groups of viruses have diverged in a distant past, potentially during the split of Sulfolobales and Thermoproteales from their common ancestor. Due to the shared virion organization, we suggest unifying *Tristromaviridae* and the order *Ligamenvirales* within a class ‘Tokiviricetes’ (*toki* means ‘thread’ in Georgian [
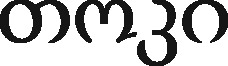
] and *viricetes* is an official suffix for a virus class), the first class-rank taxon for archaeal viruses. More importantly, our results bring to light another component of the structural ‘dark matter’ of the global virosphere and suggest that the ultimate knowledge of the complete set of structural folds used by viruses for virion formation might be within reach.
